# Assessment of a Potential Synergistic Effect of Souvenaid^®^ in Mild Alzheimer’s Disease Patients on Treatment with Acetylcholinesterase Inhibitors: An Observational, Non-Interventional Study

**DOI:** 10.3233/JAD-201357

**Published:** 2021-04-20

**Authors:** Félix Viñuela, Angeles Barro

**Affiliations:** a Instituto Neurológico Andaluz, Hospital Victoria Eugenia, Sevilla, Spain; b Unidad Deterioro Cognitivo, Hospital Universitario Virgen Macarena, Sevilla, Spain

**Keywords:** Alzheimer’s disease, cognitive dysfunction, Fortasyn Connect, observational study, pragmatic clinical trials

## Abstract

We evaluated the efficacy and safety of Souvenaid (a multinutrient supplement) in patients with mild Alzheimer’s disease (AD) in real clinical practice and assessed a potential synergistic effect of acetylcholinesterase (AChE) inhibitors. Clinical Dementia Rating (CDR) scale was evaluated after six months follow-up. Patients were divided into 4 groups according to the treatment they received: Souvenaid + AChE inhibitors (*n* = 23); only Souvenaid (*n* = 8); only AChE inhibitors (*n* = 7); no treatment (*n* = 16). The Souvenaid + AChE inhibitors and Souvenaid alone groups were associated with significantly lower increases in CDR per month than the AChE inhibitors or no treatment ones. The efficacy of Souvenaid + AChE inhibitors tended to be higher than Souvenaid alone.

## INTRODUCTION

Nutritional factors can alter the risk of developing Alzheimer’s disease (AD) and its rate of progression. There is increasing interest in nutrition as a modifiable risk factor for the disease [1].

Souvenaid is a daily medical supplement that contains Fortasyn Connect, a multinutrient combination developed to specifically address AD nutritional deficiencies. It consists of docosahexaenoic acid, eicosapentaenoic acid, uridine monophosphate, choline, phospholipids, selenium, folic acid, and vitamins B12, B6, C, and E [2]. Randomized controlled trials results demonstrated that Souvenaid is well tolerated, has an effect on brain functional connectivity, and improves memory performance in drug-naïve pat-ients with mild AD [3–9]. In addition, Souvenaid prevents the loss of cognition and function and reduces the rate of hippocampus atrophy and total brain volume in patients with prodromal AD [8, 9].

Additionally, it was also demonstrated that acetylcholinesterase (AChE) plays an important role in amyloid-β aggregation during the early stages of senile plaque formation in AD. Cholinesterase inhibitors studies showed no benefit on cognitive outcomes or progression reduction from mild cognitive impairment (MCI) to dementia, although some studies could not exclude an important effect [10]. These drugs are often prescribed for patients with MCI [11].

In this work, we aimed to determine Souvenaid’s efficacy and safety in patients with mild AD in real clinical practice, and to elucidate a potential synergistic effect of combined therapy with Souvenaid plus AChE inhibitors on this population.

## MATERIALS AND METHODS

This was a prospective, non-interventional study that was conducted between May 2017 and June 2018 in one single center. Sixty consecutive patients with a diagnosis of mild AD and a Mini-Mental State Examination (MMSE) score of≥20 were recruited. For the diagnosis of dementia, the NIA-AA (National Institute of Aging Alzheimer’s Association) 2011 criteria for probable AD were followed. For its graduation as mild cognitive decline or mild dementia, the Reisberg Global Deterioration Scale was followed with a score equal to 3 or 4 respectively. All patients were offered an AChE inhibitor treatment, unless this was contraindicated. Similarly, Souvenaid was offered to all patients, and they were free to follow the relevant recommendations. Six months after the inclusion in the study, patients were analyzed considering the therapies they had followed: Souvenaid plus AChE inhibitors, only Souvenaid, only AChE inhibitors, or neither Souvenaid nor AChE inhibitors.

Together with baseline clinical and demographic characteristics, the main variable analyzed was the Clinical Dementia Rating (CDR) [12] scale, which was evaluated at baseline and after six months of follow-up. The CDR is a 5-point scale used to characterize six domains of cognitive and functional per-formance applicable to AD (Memory, Orientation, Judgment and Problem Solving, Community Affairs, Home and Hobbies, and Personal Care). Higher scores reflect higher cognitive impairment. The variables used to assess treatment efficacy over time were the change in the CDR scale sum of boxes (CDR-SB) scores at the end of the follow-up, and CDR-SB changes by months of therapy. Adherence and side-events were registered at the end of the follow-up. Adherence to Souvenaid was considered good if participants consumed 90% or more of the product for more than 90% of the study duration. The Eth-ics Committee for Clinical Research approved the protocol. Informed consent was obtained from all participants.

Continuous data were presented as mean±standard deviation or median and interquartile range (IQR). Qualitative variables were presented as percentages. Continuous variables were analyzed using parametric or nonparametric tests when appropriate, after the normality of the data was evaluated. Multiple lineal regression models were used to determine baseline variables independently related to changes in CDR-SB at the end of the follow-up, and to changes in CDR-SB per month.

## RESULTS

A total of 60 participants were recruited in the study. Their demographic and clinical characteristics are shown in Table 1. Of this population, 54 (90%) completed the 6-month follow-up and their data were available for analysis. At the end of the follow-up, the patients were divided into 4 groups according to the treatment they had followed: Souvenaid and AChE inhibitors (Group 1: *n* = 23); only Souvenaid (Group 2; *n* = 8); only AChE inhibitors (Group 3; *n* = 7); neither Souvenaid nor AChE inhibitors (Group 4; *n* = 16).

**Table 1:** Demographic and clinical characteristics

n	60
Female, n (%)	37 (61.7)
Mean age (±SD); y	75.8±9.0
Age range; y	55–94
Median time from diagnosis (IQR); months	15 (8–26)
Median duration of follow-up (IQR); months	6.3 (2.7–9.8)
Treatment with Souvenaid, *n* (%)	34 (56.7)
Treatment with cholinesterase inhibitors, *n* (%)	35 (58.3)
Treatment with memantine, *n* (%)	11 (18.3)
Global Deterioration Scale score, *n* (%)
Mild cognitive decline (GDS = 3)	24 (40)
Mild dementia (GDS = 4)	36 (60)
Baseline median CDR-SB score (IQR)	3 (2.5–4.0)
Comorbidities, n (%)
Depression	25 (41.7)
Cardiovascular risk factors	16 (26.7)
Cerebrovascular disease	9 (15.0)
Chronic pulmonary disease	4 (6.7)
Parkinson’s disease/Parkinsonism	4 (6.7)

CDR-SB, Clinical Dementia Rating - Sum of Boxes; IQR, interquartile range; SD, standard deviation.

A total of 6 patients were excluded from the study (10%), because they were lost to follow-up: 2 patients from Group 1 (Souvenaid + AChE inhi-bitors), 1 patient from Group 2 (only Souvenaid), and 3 patients from Group 3 (Only AChE inhibitors). All participants tolerated Souvenaid well except of one, who did not like the taste. There were no major complications related to the supplementation or the AChE inhibitor therapy. All patients who completed the follow-up reported good adherence to Souvenaid and ACH inhibitors. All patients, who did not take Souvenaid mentioned financial reasons, with the exception of the patient who did not tolerate it because of the taste.

There were no significant baseline differences between groups in terms of age, duration of AD, or presence of neurologic, or psychiatric morbidity. The proportion of males was higher in Group 3. Median CDR-SB score at baseline was 3 (2.5–4), with no significant differences between groups except between Group 1 and 4 (3.5 versus 2.5; *p* = 0.021), indicating higher cognitive impairment in Group 1.

### Changes in CDR-SB scores at the end of the follow-up

**Fig. 1:**
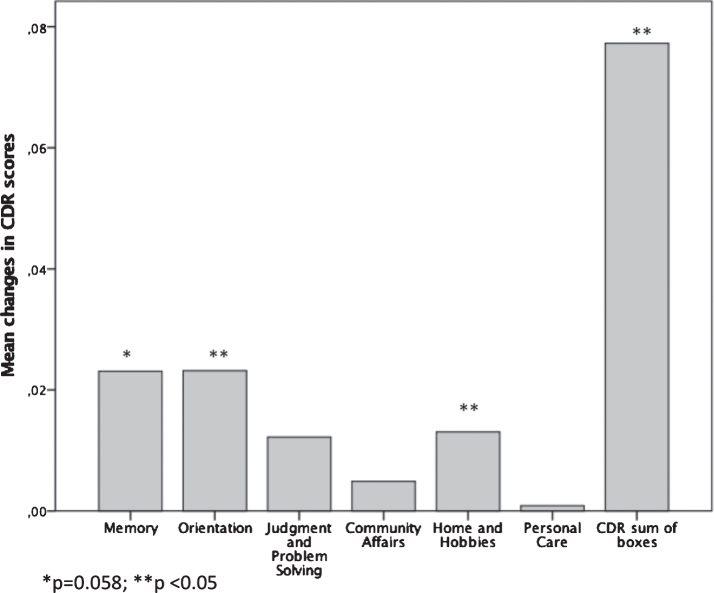
Mean changes in CDR scores and in CDR sum of boxes.

After 6-month follow-up, there was a statistically significant mean CDR-SB scores (*p* < 0.001), mean CBR domains related to Orientation (*p* = 0.03) and Home and Hobbies (*p* = 0.025) increase. Memory was in the limit of reaching significance (*p* = 0.058) (Fig. 1). There were statistically significant differences in the changes of the CDR-SB score at the end of the follow up in patients from Group 1 (Souvenaid + AChE inhibitors) as compared with patients from Group 4 (no treatment) (*p* = 0.003); whereas there were no differences between Group 2 (only Souvenaid) or Group 3 (Only AChE inhibitors) as compared with Group 4 (no treatment) (*p* = 0.094 and *p* = 1.0, respectively). There was a correlation between the increments in CDR-SB scores and age (r = 0.282; *p* = 0.039). CDR-SB scores increments tended to be higher in males (*p* = 0.10), but they were not associated to other variables such as duration of AD, co-morbidities, or use of memantine.

In a multiple lineal model, including as indepen-dent variables age, sex, and the four treatment categories, the use of Souvenaid + AChE inhibitors (Group 1) remained the only variable associated to lower CDR-SB changes at the end of the follow-up (R^2^: 0.248; *p* = 0.0029). Similarly, in a model that included the four treatment categories and baseline patient co-morbidities, the use of Souvenaid + AChE inhibitors remained the only statistically significant factor associated to lower CDR-SB changes at the end of the follow-up (R^2^: 0.16; *p* = 0.043).

### Changes in CDR-SB scores per month of treatment

**Fig. 2:**
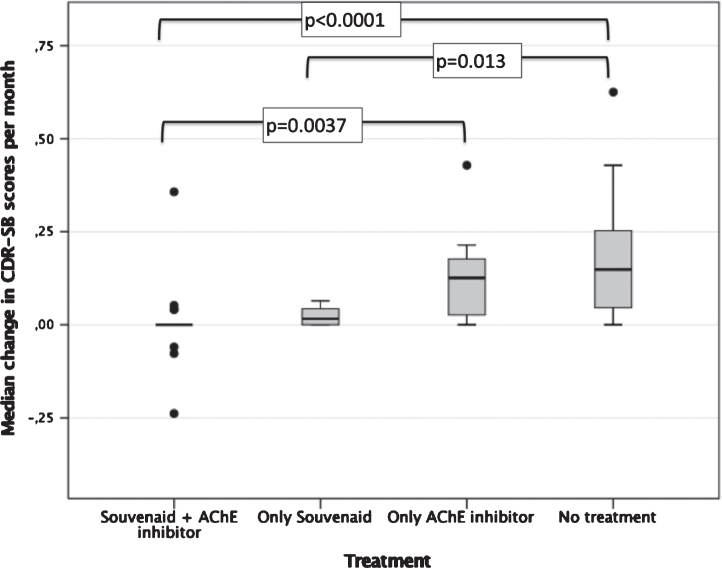
Median monthly changes in CDR-SB scores according to treatment group.

Mean monthly increase in CDR-SB score was 0.0773±0.14 points. Monthly increases in CDR-SB scores were significantly lower in patients on treatment with Souvenaid + AChE inhibitors (Group 1) in comparison with those treated only with AChE (Group 3; *p* = 0.0037) or no treatment (Group 4; *p* < 0.0001). Similarly, increments were lower in patients on treatment only with Souvenaid (Group 2) versus no treatment (Group 4; *p* = 0.013; Fig. 2). There was no significant correlation between monthly CDR-SB scores changes and other variables, including age, sex, duration of AD, co-morbidities, or use of memantine, although increments tended to be higher in older people (r = 0.217; *p* = 0.11)

Interestingly, the monthly increase in CDR-SB scores tended to be lower in patients on treatment with Souvenaid + AChE inhibitors (Group 1) as compared with those on treatment with Souvenaid only (Group 2; *p* = 0.14; Fig. 2). Furthermore, monthly increment of CDR-SB scores was lower in Group 2 (only Souvenaid) as compared with Group 3 (only AChE inhibitors), with differences at the limit of reaching significance (*p* = 0.094) (Fig. 2).

In a multiple lineal model, including as indepen-dent variables age, sex and the four treatment categories, the use of Souvenaid + AChE inhibitors (Group 1) or the use of Souvenaid (Group 2) were significantly associated to lower monthly CDR-SB scores changes (R^2^ = 0.249; *p* = 0.029). Age and sex did not contribute to the model. In a second model that included the four treatment categories together with the morbidities, groups 1 and 2 (Souvenaid + AChE inhibitors or only Souvenaid) were associated to lower changes in CDR-SB scores per month (R^2^ = 0.258; *p* = 0.014).

Regarding the different domains of the CDR scale, the treatment with Souvenaid (Groups 1 and 2) seemed to benefit numerically more Memory and Orientation domains (Supplementary Figure 1).

## DISCUSSION

In this observational study conducted in real life conditions, the treatment with Souvenaid for six months, alone or in combination with AChE inhi-bitors, was well tolerated and seemed to be associated to less cognitive impairment deterioration than the treatment only with AChE inhibitors or no treatment in patients with mild AD. This results are important since there are no pharmacologic agents approved by the Food And Drug Administration (FDA) for the treatment of MCI [10].

Growing evidence from epidemiological and clinical studies showed that nutritional factors can influence AD risk and its rate of clinical progression [13]. Patients with early AD may have a poor nutrient status to support the formation of neuronal membrane components [14]. Souvenaid is an oral mul-tinutrient supplement with a favorable safety profile and is associated with clinically detectable effects in patients with early AD [15]. Preclinical studies results suggested that this supplementation provides precursors and cofactors that are necessary to form neuronal membranes and might support the synthesis of new synapses and the maintenance of existing ones [15]. The effects of Souvenaid on cognition and memory performance in patients with AD were evaluated in three double-blind, multi-center, randomized, controlled clinical trials [3–5]. These studies showed that Souvenaid increased the availability of nutrients needed to support the formation of phospholipids and to maintain neuronal membrane integrity, and improved memory performance [15]. Number needed to treat (NNT) analysis from clinical trial suggests that for every six patients taking Souvenaid one will achieve a clinically detectable memory performance benefit [15]. Although the primary endpoints of these clinical trials differ from our study, our results, based on real clinical practice, are consistent with the conclusion that Souvenaid is associated with clinical benefits in patients with mild AD. In addition, a recent randomized controlled trial conducted in prodromal AD, showed positive CDR-SB scores results associated with the use of Souvenaid for 36 months, supported by other measures of cognition, function, and brain atrophy, including some that appeared only after long-term intervention [8, 9].

Regarding the benefit of the addition of AChE inhibitors to Souvenaid, our results were not concl-usive, but a possible synergistic effect cannot be entirely ruled out. In the bivariate analysis and in the multiple lineal models, only the combination Souvenaid + AChE inhibitors, and not Souvenaid alone, was consistently associated with lower declines in CDR-SB scores (at the end of the follow-up and when considering monthly changes). However, the use of Souvenaid alone seemed to be superior, at least compared to the group with no treatment, in the lower decline in median CDR-SB scores per month (Fig. 2), and remained an independent factor associated to lower decline in CDR-SB scores per month in the multiple lineal models. In a direct comparison between Souvenaid + AChE inhibitors and Souvenaid alone, monthly increment of CDR-SB scores tended to be lower in patients on treatment with Souvenaid + AChE inhibitors (Group 1) in comparison with those on treatment only with Souvenaid (Group 2; *p* = 0.14). These data suggest that the addition of AChE inhibitors to Souvenaid might have some slight synergistic effect. However, given the limited number of patients in the group treated only with Souvenaid, it is necessary to include a higher number of patients into this group to consistently reach this conclusion. In conclusion, in our study clinical benefit seemed to be exerted mostly by Souvenaid and the addition of AChE inhibitors did not seem to worsen the positive results.

Our study has certain limitations, such as the short duration of the follow-up or the reduced number of patients in some of the study groups. However, despite the limited number of patients, it seems that there are large differences between groups in terms of clinical benefit, at least between groups 1 and 4 (Souvenaid + AChE inhibitors versus no treatment). The fact that the study was conducted in nearly real life conditions may support the conclusions; nevertheless long-term studies are necessary to evaluate whether the clinical benefit of Souvenaid±AChE inhibitors is sustainable in the long-term. Positive data regarding the effects of Souvenaid on prodromal AD after 36 moths of use [9] suggest that the clinical benefit of Souvenaid±AChE inhibitors may become greater after long-term use. In addition, we analyzed AChE inhibitors in general. It is possible that clinical differences exist among different AChE inhibitors. However, no particular AChE inhibitor seems to be superior to the others in terms of clinical benefit [16].

As a conclusion, Souvenaid is an oral multinutrient supplement that is well tolerated and shows benefit on domains of cognition in patients affected by mild AD. Souvenaid efficacy in terms of prevention of cognitive impairment seems to be superior to the treatment with AChE inhibitors or no treatment in this population. The addition of AChE inhibitors to Souvenaid does not reduce the positive results of Souvenaid and might have some limited synergistic effect. Additional research is required to evaluate tolerability and clinical benefit during long-term management.

## Supplementary Material

Supplementary Material
