# Supplementary material for: Assessment of a Potential Synergistic Effect of Souvenaid® in Mild Alzheimer’s Disease Patients on Treatment with Acetylcholinesterase Inhibitors: An Observational, Non-Interventional Study
Source: J Alzheimers Dis. 2021 Apr 20;80(4):1377–82. doi: 10.3233/JAD-201357 (PMC8150480; doi:10.3233/JAD-201357)

Supplementary Material

Assessment of a Potential Synergistic Effect of Souvenaid® in Mild Alzheimer's Disease Patients on Treatment with Acetylcholinesterase Inhibitors: An Observational, Non-Interventional Study

Supplementary Figure 1. Mean monthly changes in CDR scores according to treatment groups.

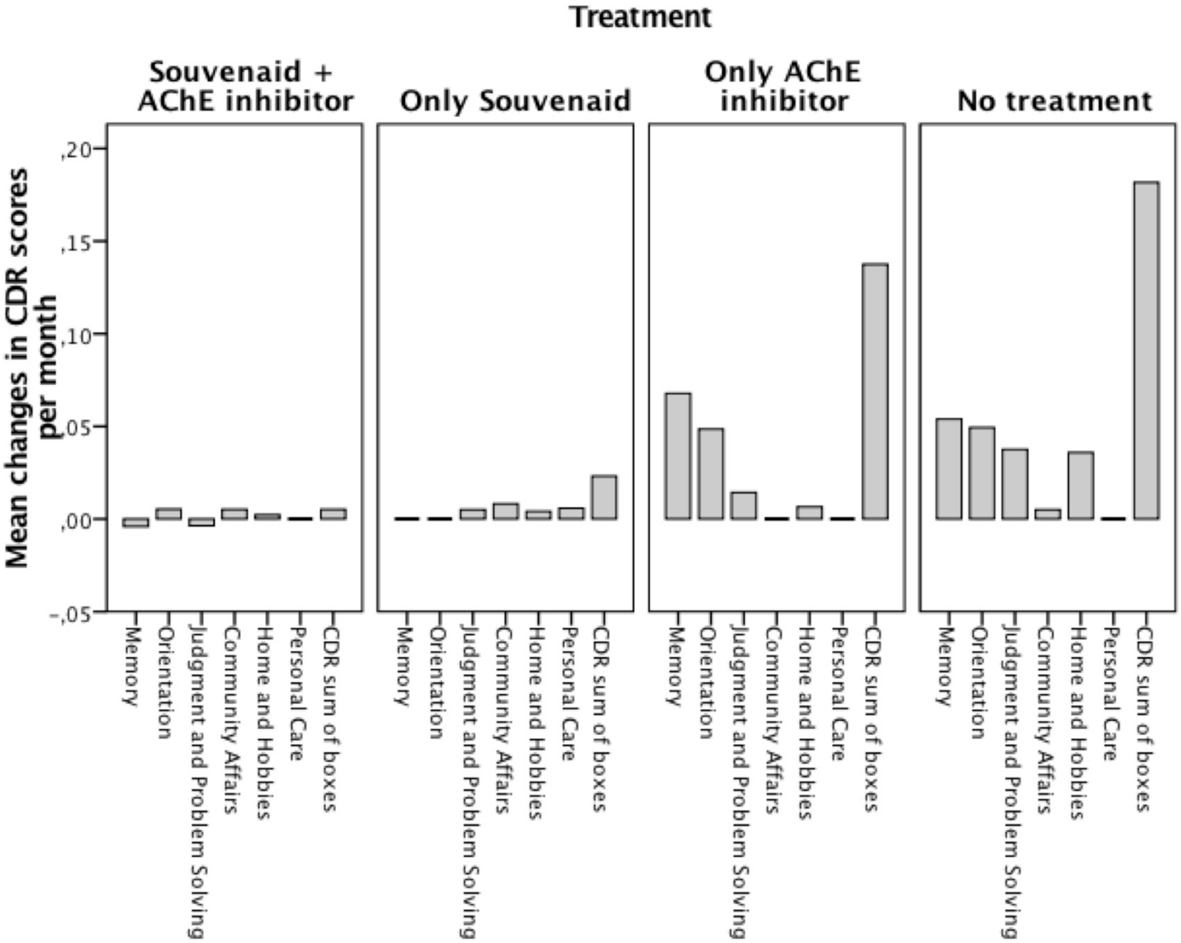

Supplement: Supplementary Material [file jad-80-jad201357-s001.pdf]
